# Etiology of genetic muscle disorders induced by mutations in fast and slow skeletal MyBP-C paralogs

**DOI:** 10.1038/s12276-023-00953-x

**Published:** 2023-03-01

**Authors:** Taejeong Song, Maicon Landim-Vieira, Mustafa Ozdemir, Caroline Gott, Onur Kanisicak, Jose Renato Pinto, Sakthivel Sadayappan

**Affiliations:** 1 Division of Cardiovascular Health and Disease, Department of Internal Medicine, University of Cincinnati, Cincinnati, OH 45267 USA; 2 Department of Biomedical Sciences, Florida State University College of Medicine, Tallahassee, FL 32306 USA; 3 Department of Pathology and Laboratory Medicine, College of Medicine, University of Cincinnati, Cincinnati, OH 45267 USA

**Keywords:** Translational research, Cardiac hypertrophy

## Abstract

Skeletal muscle, a highly complex muscle type in the eukaryotic system, is characterized by different muscle subtypes and functions associated with specific myosin isoforms. As a result, skeletal muscle is the target of numerous diseases, including distal arthrogryposes (DAs). Clinically, DAs are a distinct disorder characterized by variation in the presence of contractures in two or more distal limb joints without neurological issues. DAs are inherited, and up to 40% of patients with this condition have mutations in genes that encode sarcomeric protein, including myosin heavy chains, troponins, and tropomyosin, as well as myosin binding protein-C (*MYBPC*). Our research group and others are actively studying the specific role of *MYBPC* in skeletal muscles. The *MYBPC* family of proteins plays a critical role in the contraction of striated muscles. More specifically, three paralogs of the *MYBPC* gene exist, and these are named after their predominant expression in slow-skeletal, fast-skeletal, and cardiac muscle as sMyBP-C, fMyBP-C, and cMyBP-C, respectively, and encoded by the *MYBPC1, MYBPC2*, and *MYBPC3* genes, respectively. Although the physiology of various types of skeletal muscle diseases is well defined, the molecular mechanism underlying the pathological regulation of DAs remains to be elucidated. In this review article, we aim to highlight recent discoveries involving the role of skeletal muscle-specific sMyBP-C and fMyBP-C as well as their expression profile, localization in the sarcomere, and potential role(s) in regulating muscle contractility. Thus, this review provides an overall summary of *MYBPC* skeletal paralogs, their potential roles in skeletal muscle function, and future research directions.

## Muscle disease: understanding different forms of a key muscle protein

Contraction and relaxation of both heart and skeletal muscles are regulated by different forms of a protein called MyBP-C, mutations in this protein being important causes of heart and skeletal muscle diseases. Although the cardiac form has been well studied, the two skeletal forms are not well understood. Taejeong Song and Sakthivel Sadayappan at the University of Cincinnati, USA, have reviewed current understanding of the structure and function of MyBP-C proteins, and links between mutations and disease. They report that mutations in the cardiac form are the most common cause of hypertrophic cardiomyopathy, disease of the heart muscle. Mutations in skeletal MyBP-C are associated with muscle diseases such as distal arthrogryposes, contracture hands and feet that stiffens joints and reduces mobility. Further study of MyBP-C function may help in developing gene therapies.

## Introduction

**Fig. 1:**
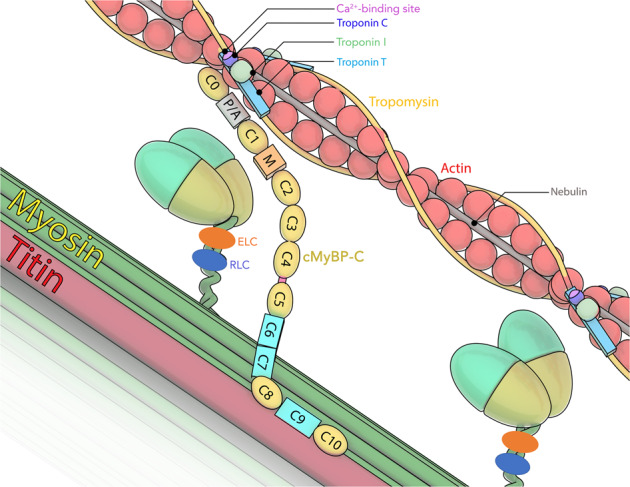
Diagram of cMyBP-C interactions with thick and thin filaments in sarcomeres. The C’-region (C8-C10) anchored in light meromyosin and titin localizes and stabilizes the MyBP-C protein. The N’-region (C0-C2) binds to either the myosin head or actin/tropomyosin and regulates the kinetics of actomyosin interaction and calcium sensitivity.

The sarcomere is the smallest functional unit of contraction and the primary component of striated muscle, accounting for over 90% of its protein volume, and acts as a building block of muscles. Proper contraction and relaxation cycles of the sarcomere are critical for the functions of cardiac and skeletal muscles, such as pumping blood out of the heart and maintaining body posture and locomotion, respectively. Thick and thin filament proteins, such as myosin and actin, comprise the two well-defined components in the sarcomere that generate mechanical force by utilizing the energy produced from adenosine triphosphate (ATP) metabolism. However, other sarcomere proteins, including myosin binding protein-C (MyBP-C), are also essential for the steadfast function of striated muscle contractility. Among multiple structural regions of the sarcomere, the C-zone of the A band is the region wherein actin and myosin overlap and form cross-bridges (Fig. 1). MyBP-C, encoded by the MYBPC gene, is specifically localized to the C-zone and regulates the contact of actin and myosin. To date, three MyBP-C paralogs have been identified. Cardiac MyBP-C (cMyBP-C, encoded by *MYBPC3*) is exclusively expressed in the heart, whereas the other two isoforms, slow and fast MyBP-C (sMyBP-C and fMyBP-C, encoded by *MYBPC1* and *MYBPC2*, respectively), are predominantly expressed in skeletal muscle^1^. Studies defining the structure and function of MyBP-C in the sarcomere have shed light on the molecular mechanisms underlying the fine-tuning of muscle contraction and relaxation and on the processes and prognoses of cardiomyopathies and skeletal diseases induced by MYBPC gene mutations.

MyBP-C was first discovered in 1971 from rabbit skeletal muscle tissue during myosin filament isolation^2^. Since then, little progress has been made until the late 1990s, when multiple *MYBPC3* mutations were found in patients with hypertrophic cardiomyopathy (HCM)^3,4^. Over the last two decades, more than 500 *MYBPC3* mutations have been found, and the association of *MYBPC3* mutations with developing HCM and heart failure (HF) has been intensively studied^5–7^. After the generation of the first knock-in mouse model with the carboxyl-terminal mutation of *MYBPC3* developed by the Seidman group in 1999, studies have shown that ablation of cMyBP-C resulted in severe dilated cardiomyopathy^8^. A similar outcome was observed by the Robbins group in 1999 through the transgenic overexpression of cMyBP-C truncated proteins in mice^9^. These studies determined the critical role of the carboxyl terminus of cMyBP-C in the development of cardiomyopathies. Additionally, two different knockout (KO) mouse models from two separate laboratories, the Moss laboratory^10^ and the Carrier laboratory^11^, independently determined the critical role of cMyBP-C in cardiac function. Moreover, cMyBP-C has multiple phosphorylation sites in its N-terminus, and its binding affinity to the myosin head is distinctly dependent on the phosphorylation status^12,13^. cMyBP-C phosphorylation has been shown to regulate the speed of muscle contraction via actomyosin interactions^14^. However, pathological outcomes arise if the level of cMyBP-C phosphorylation is reduced, e.g., cardiomyopathies and HF, implying decreased contractile function^15,16^. Constitutive cMyBP-C phosphorylation, however, improves cardiac function and has cardioprotective effects after ischemic injury^17^. Moreover, upon acute cardiac injury, a cleaved N-terminus of cMyBP-C is released into the bloodstream and was thus shown to be a potential biomarker of a compromised heart^18–21^. A recent study found that cMyBP-C phosphorylation might regulate calcium transients and sensitivity via direct contact with tropomyosin to enhance binding between calcium and troponin^22^. Other recent studies have focused on determining the role of the amino terminus region of cMyBP-C^23^. Overall, these structure-activity studies have further characterized cMyBP-C with a particular focus on the importance of its phosphorylation state.

Compared with those on cMyBP-C, studies on skeletal MyBP-C isoforms (slow and fast MyBP-C) are not as robust, despite the revelations of recent reports showing that mutations in skeletal MyBP-C are associated with inherited skeletal muscle diseases, such as distal arthrogryposis (DA) and lethal congenital contracture syndrome (LCCS)^24,25^. Nevertheless, multiple phosphorylation sites of sMyBP-C have been identified, and the functional roles of sMyBP-C and fMyBP-C have continued to emerge recently^26–28^. Thus, in this article, we review the basic structure and function of MyBP-C and diseases linked to its mutations. We also discuss possible therapeutic approaches by targeting MyBP-C for the treatment of cardiac and skeletal muscle diseases.

## Structure and expression of MYBPC paralogs

**Fig. 2:**
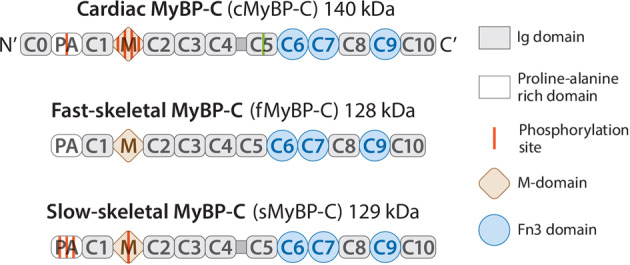
Domain structure of the 3 isoforms of MyBP-C. The three isoforms of MyBP-C – cardiac, fast-skeletal, and slow-skeletal – all contain seven immunoglobulin domains (Ig), three fibronectin 3 domains (Fn3), an M-domain, and a proline and alanine-rich region. The linker (horizontal line) between immunoglobulin domains C4 and C5 has been reported in both cardiac and slow-skeletal isoforms of MyBP-C. Top: The cardiac isoform (*MYBPC3* gene) has an additional Ig domain at the N-terminus, 28 residual inserts in the C5 domain (green vertical line), one phosphorylation site in the proline- and alanine-rich region, and four phosphorylation sites in the M-domain. Middle: The fast-skeletal isoform of MyBP-C (*MYBPC2* gene) has no reported phosphorylation sites. Bottom: The slow-skeletal isoform of MyBP-C (*MYBPC1* gene) has three phosphorylation sites in the proline- and alanine-rich region and one site in the M-domain.

MyBP-C is approximately 40 nm long and 3 nm wide with a molecular weight of ~140 kDa^29–33^. Interestingly, these three paralogs share similar core structural features formed by a linear series of globular domains, including seven immunoglobulin-like and three fibronectin-like domains depicted as C1-C10 from the N-terminus, an additional M-domain linking C1 and C2, and a proline/alanine-rich sequence (PA region) preceding the C1 domain (Fig. 2). Importantly, the cMyBP-C isoform differs from skeletal isoforms with three additional features. First, the cardiac isoform contains an additional immunoglobulin module (C0 domain) at the N-terminus. Second, it has a 28-residue-long loop insertion within the C5 immunoglobulin domain. Finally, it has four phosphorylation sites in the M-domain and a novel phosphorylation site within the PA region^14,34^. In contrast, the sMyBP-C isoform has one phosphorylation site within the M domain and three phosphorylation sites in the PA region, whereas no phosphorylation site has been reported for fMyBP-C^26^. The phosphorylation sites of cMyBP-C within the M domain include serine residues at positions 273, 282, 302, and 307 in mice that are the targets of many kinases, including protein kinase A, C and D (PKA, PKC, and PKD, respectively), and calcium/calmodulin-dependent kinase-II (Table 1 and Fig. 2)^12,35,36^. GSK3β phosphorylates serine 133 within the PA region of cMyBP-C^34^. In addition to phosphorylation, redox modifications of cMyBP-C have also been reported in different disease models of HF. In contrast to phosphorylation, redox modifications occur through nonenzymatic reactions. These redox modifications include oxidative stress-induced carbonylation, S-nitrosylation, and S-glutathionylation^37,38^.

**Table 1:** Expression, modification and related diseases of three MyBP-C paralogs.

	sMyBP-C	fMyBP-C	cMyBP-C
Gene code and chromosome location in human (mouse)	*MYBPC1* Chromosome 12 (10)	*MYBPC2* Chromosome 19 (7)	*MYBPC3* Chromosome 11 (2)
mRNA and protein size (mouse)	3941 bp and 1127 aa	3610 bp and 1136 aa	4224 bp and 1270 aa
Primary expression tissue	Skeletal and smooth muscles	Fast twitch skeletal muscle	Cardiac muscle
Phosphorylation sites	S59, S62, T84, S204^26,109^	None	S133, S273, S282, S302, S307^13,34^
Pathogenic mutations	W236R, Y247H, E248K, L259P, L263R, R318X, P319L, E359K, Y856H^24,114,115^	T236I, S255T, V307A, F510fs, A1065V^110^	>500
Related diseases	Distal arthrogryposis type 1b, Lethal congenital contracture syndrome 4, Congenital myopathy with tremor	Unclassified distal arthrogryposis	HCM DCM Left ventricular noncompaction
Phenotypes of knockout animal models	Reduced survival, blunted motor response and severe body curvature (zebrafish)^116^ Severe functional loss (mouse)^28^	Reduced force generation, disrupted sarcomere structure and muscle atrophy (zebrafish)^117^ Reduced muscle function, calcium sensitivity and regenerative capacity (mouse)^27^	Severe HCM and DCM (mouse)^102^

Each *MyBP*-C paralog is encoded in different chromosomes, and the genes are expressed in specific tissues and have distinct phosphorylation profiles. Mutations of each paralog are related to the development of congenital skeletal and cardiac diseases. NCBI Gene ID: *Mybpc1* (109272), *Mybpc2* (233199), *Mybpc3* (17868).

Despite the suggestive names and their predominant expression patterns that place sMyBP-C and fMyBP-C with skeletal muscles and cMyBP-C exclusively with cardiac tissue^39^, relatively small, but significant, amounts of sMyBP-C and fMyBP-C are expressed within the heart. In fact, studies have demonstrated that sMyBP-C is expressed in the mammalian heart (i.e., atrium and interatrial septum) and that fMyBP-C is expressed in the heart in murine models of HF^40,41^. Moreover, even cMyBP-C is expressed in the developing skeletal muscles of chicken embryos^42^. However, cMyBP-C expression remains restricted to the cardiac muscles of mammalian embryos^43^.

Although fMyBP-C can be detected at very low levels within slow twitch muscle, it is almost exclusively expressed in fast twitch muscle, specifically within type IIb fibers^26,41^. Moreover, sMyBP-C is not restricted to slow twitch muscles because it is also abundantly expressed within fast twitch muscles^27^. Moreover, fMyBP-C and sMyBP-C can be coexpressed within the same muscle types and even coexpressed within the same sarcomere^44–47^. Additionally, multiple sMyBP-C variants have been described as expressed in differing amounts in both slow twitch and fast twitch muscles^48^. In fact, sMyBP-C undergoes a higher level of splicing compared with fMyBP-C and cMyBP-C. At least 14 protein-coding transcripts have been reported in humans and mice that result in sMyBP-C variants with an approximate range between 126 and 132 kDa^48,49^. Much of this alternative splicing occurs within the first six exons encoding the PA region, although alternative splicing can also occur in regions encoding the M-domain, C7 domain, and carboxyl terminus. Additionally, sMyBP-C variants can be differentially expressed in different muscle groups and even within different myofibers of the same muscle^28,48^.

Each MyBP-C paralog is known to follow a different spatiotemporal expression pattern. In particular, cMyBP-C is expressed early in embryonic development together with titin and myosin, whereas skeletal muscle paralogs are expressed at later stages of development after the completion of titin and myosin expression, and sMyBP-C expression precedes that of fMyBP-C^1,50,51^.

## Role of MYBPC in cardiac and skeletal muscle function

Based on their phosphorylation status, as suggested previously, sarcomere proteins, including MyBP-C, play key regulatory roles in striated muscle contraction and cardiac hemodynamics^52,53^. MyBP-C isoforms contain multiple phosphorylation sites targeted by different kinases, such as PKA, PKC, PKD, CaMK, and ribosomal S6 kinase^12,18,54–56^. cMyBP-C is particularly unique because it has a flexible phosphorylation motif that is not present in slow and fast skeletal MyBP-C (Fig. 2). This motif is located at the N-terminal domain and targeted by PKA and CAMK under β-adrenergic stimulation in live myocardium^12,57^. In sMyBP-C, PKA has been reported to phosphorylate serine 59 and serine 62, whereas PKC phosphorylates serine 83 and threonine 84. Serine 204 is phosphorylated by both PKA and PKC^26,58^. It has been almost 40 years since the first description of cMyBP-C M domain phosphorylation in intact amphibian myocardium^59^. Since then, many in vivo and in vitro models and studies have provided more insight into the molecular mechanisms underlying muscle regulation by MyBP-C phosphorylation.

**Fig. 3:**
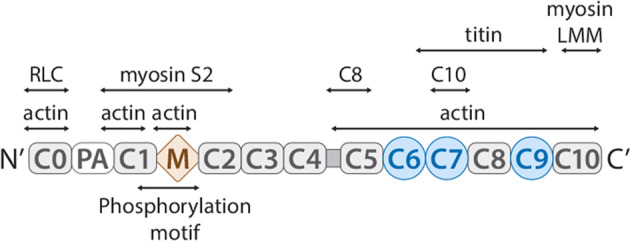
cMyBP-C binding partners and modifications. C-terminal region (C7-C10) is anchored in titin and meromyosin (myosin LMM) and stabilizes the expressed protein. N-terminal (C0-C2) is a functional region that interacts with actin and myosin (S2 and RLC) in a manner dependent on the phosphorylation state and regulates muscle contraction and relaxation.

A previous study utilizing skeletal muscle reconstituted with soluble recombinant MyBP-C fragments demonstrated that the presence of the unphosphorylated MyBP-C motif led to a decrease in Ca^2+^-activated isometric maximal force, an increase in myofilament Ca^2+^-sensitivity and maximal rigor force and acceleration of the development of rigor force and rigor stiffness^60^. All these results were eliminated by PKA-dependent phosphorylation of the MyBP-C motif, revealing that the cycling of myosin heads is modulated by the MyBP-C regulatory domain in a phosphorylation-dependent manner. Since the C-terminal domain-dependent anchorage of MyBP-C to the thick filament is not required to obtain such results, it has been suggested that the mechanism of MyBP-C regulation is at least partly independent of a tether^60^. More recently, this idea was confirmed by a study that used a soluble C1-C2 motif in permeabilized myocytes from wild-type and cMyBP-C-KO mice. The phosphorylation of C1-C2 by PKA reduces the ability of MyBP-C to directly increase myofilament Ca^2+^ sensitivity. These results demonstrate that the C1-C2:S2 interaction alone is sufficient to disturb the contractility and myofilament Ca^2+^ sensitivity in a manner independent of a tethering mechanism^61^. McClellan and colleagues presented an inverse relationship between the percentage of unphosphorylated protein and Ca^2+^-activated maximal force in which a higher dephosphorylation level corresponds to lower force production^62^. These findings support a previous study revealing that MyBP-C motif phosphorylation allows myosin heads to extend outward from the thick filament backbone^63–68^. In vitro studies have shown that the MyBP-C N-terminal domain interacts with actin and/or myosin^69–71^. The interaction between the C1-C2 domain and the myosin S2 subdomain (S2) is a phosphorylation-dependent process in which phosphorylated cMyBP-C no longer interacts with S2 but rather moves toward the thin filament to bind actin and thus regulate contractility^60,72–76^. In addition, the C0 domain of cMyBP-C interacts with myosin regulatory light chain (RLC), which has been verified by multiple in vitro experiments, and HCM-related C0 domain mutations (R35W and K87E) have been shown to reduce the binding affinity of C0 to myosin RLC^70^. Therefore, the N-terminal domain of cMyBP-C modulates force production and contractile mechanics by regulating the actin-myosin interactions (Fig. 3)^77–81^.

Cardiomyocyte-specific transgenic mice were generated utilizing the α-myosin promoter to express constitutively active phosphorylated cMyBP-C in which serines 273, 282, and 302 were mutated to aspartic acid^17^. This chronic phosphorylation causes subtle changes in the sarcomere ultrastructure and an irregular steric arrangement of the thick filament lattice^17^. In contrast, transgenic mice carrying cMyBP-C, in which the phosphorylation sites are modified to express non-phosphorylatable alanines, show a reduced cardiac contractility, an altered sarcomeric structure and upregulation of transcripts linked to the hypertrophic response^18^. These studies show how unbalanced levels of MyBP-C phosphorylation can affect the regulation of muscle contraction. In addition, cMyBP-C regulates contractility at the sarcomeric and organ levels, and cMyBP-C phosphorylation is harmonized with the phosphorylation states of other sarcomeric proteins, such as cardiac troponin I^82^. Similar to cMyBP-C phosphorylation, PKA-mediated sMyBP-C phosphorylation enhances actomyosin interaction by exhibiting accelerated cross-bridge cycling at all levels of Ca^2+^ activation^58^. Interestingly, a consensus has not been reached on the regulatory role of cMyBP-C relative to the cross-bridge cycling speed. For example, the results from the rat myocardium show slower cross-bridge cycling, whereas the opposite was observed in the mouse myocardium^83–85^. Despite the discrepancy, possibly from the differences in sample preparation or study methodology, it is possible to deduce that the phosphorylation of cardiac and slow MyBP-C does affect the cross-bridge kinetics. Moreover, the role of fMyBP-C in muscle regulation remains poorly studied. Recently, however, an extensive in vivo and in vitro study was performed with a homozygous *Mybpc2*-KO mouse model (C2^−/−^)^27^. The deletion of fMyBP-C led to a significant reduction in the grip strength, plantar flexor muscle strength, steady-state isometric force during Ca^2+^ activation, and myofilament calcium sensitivity. These results support previous findings in which the N-terminus of fMyBP-C enhances myofilament Ca^2+^ sensitivity and force generation^86^. Furthermore, a structural analysis assessed by small-angle X-ray diffraction of the C2^−/−^ extensor digitorum longus muscle has revealed a greater shift of myosin heads toward actin, less ordered myosin heads, and increased myofilament lattice spacing^27^. Together, these data demonstrate that fMyBP-C modulates the myofilament Ca^2+^ sensitivity, actomyosin recruitment, and cross-bridge cycling to match the myofilament contractile output to the demand and that this role is critical for sarcomere integrity, maximal speed, and force generation^27^.

The regulatory role played by MyBP-C is not restricted to thick filament regulation. A refined study performed with fixed cardiac muscle samples using super-resolution fluorescence microscopy demonstrated that the cMyBP-C N-terminal position is biased toward actin filaments in both active and relaxed muscle^76^. When a muscle is in its active state, the thin filament sliding process occurs when myosin heads are freed from the thick filament surface and allowed to move toward the thin filament to bind actin^87^. Notably, it is not necessary for myosin heads to be in an active state to be released from the thick filament^88^. For example, it has been demonstrated that the N-terminal fragment binds to the thin filament, displaces tropomyosin, locks tropomyosin toward an open conformation, activates cardiac thin filaments, and allows myosin to strongly bind to actin at low Ca^2+^ levels^22^. Upon removal of the N-terminal domain or cMyBP-C deletion, cMyBP-C loses its control over actin filament activation and sliding^10,77,78,89^. Based on its profound impact on actomyosin interactions, the absence of cMyBP-C would result in failed regulation of the bent-back structural configuration (interacting head motif, IHM) and failure to balance the energy-conserving biochemical state (super-relaxed state, SRX) adopted by myosin heads. More specifically, mice lacking MyBP-C exhibit disruption of the myosin population in the SRX state as well as weakened or abolished IHM configurations^90–92^, suggesting that MyBP-C may modulate SRX by stabilizing IHM. Although it has been shown that the cMyBP-C regulatory process is dynamically modulated by cMyBP-C phosphorylation in response to β-adrenergic stimulation, the actual regulatory mechanism remains uncertain^90,93^. To prevent actomyosin interaction, it has been proposed that the myosin heads fold onto the thick filament and that the MyBP-C N-terminal domain lies along the thick filament surface to stabilize IHM and the very low ATP turnover state of myosin^76,94–97^. A study performed with homozygous cMyBP-C-KO mice revealed a decreased myosin population in the SRX state^98^. In addition, PKA-mediated phosphorylation shifts the biochemical state of the myosin population to a disordered state (from the “OFF” to “ON” state) by reducing cMyBP-C-myosin interactions^90,95^. Craig and Padron state the following viewpoint: “Enhancement of cardiac contractility by cMyBP-C phosphorylation may result in part from depression of cMyBP-C’s stabilizing effect on the SRX, which coincides with the weakening of the IHM”^65–68^.

The distribution of MyBP-C along the different zones of the sarcomere plays an important role in understanding its regulatory control over contractility. By using single-molecule fluorescence imaging of relaxed rat soleus skeletal myofibrils, Nelson and colleagues assessed the kinetics of ATP hydrolysis in the sarcomeric C-zone, D-zone, and P-zone^93^. The myosin population located at the C-zone, the region in which MyBP-C is abundantly located, is found in the disordered relaxed (DRX) and SRX states and present a longer ATP lifetime compared with other zones that lack MyBP-C. Both the D-zone and P-zone present myosin populations predominantly in the DRX state^93^. These results confirm the notion that MyBP-C regulates the strong inhibitory state of myosin and can be considered essential for fine-tuning myofilament contractility.

Taken together, the results demonstrate that MyBP-C is a fundamental element in regulating both thin and thick filament activation, actomyosin interaction, cross-bridge kinetics, force generation, and sarcomere integrity. Perhaps it is by selective adaptation that the protein keeps myosin heads in a structurally and biochemically favorable state, according to the muscle’s demands, and thus exerts a dual regulatory effect: inhibition and activation of the striated muscle. Despite significant efforts, we still do not understand the underlying functional and structural roles of MyBP-C or how it modulates myofilament performance.

## Regulation of MYBPC and its mutations in muscle diseases

HCM is the most common form of inherited cardiomyopathy, affecting an estimated 1 in 300 individuals around the world^99^. Accumulated studies over the last two decades have shown that *MYBPC3* is the most frequently mutated gene and that it affects approximately 40% of HCM patients. The first two *MYBPC3* gene mutations at the C4-C5 linker and C9 domain were reported in patients with HCM by two different research groups in 1995, and since then, over 500 *MYBPC3* mutations have been found in HCM patients (Table 1)^3,4^. Both missense and truncated mutations in the entire *MYBPC3* genomic sequence can cause HCM by generating a dominant negative mutation or result in the production of a “poison peptide”^100,101^. It is a challenge to anticipate mutation penetrance because the binding partner of the mutant protein and additional compounding mutation in the same or different gene can influence the disease profile^46^. *Mybpc3*-KO mouse hearts show significant loss of cardiac function and structural changes within 3 days of birth. By adulthood, a significant HCM phenotype has developed in the absence of *MYBPC3*, and more than 40% of cardiac function is decreased. Although the sarcomeric microstructure is preserved, increased fibrosis and reduced calcium sensitivity are observed in *Mybpc3*-KO hearts^10,102^. Both in vivo and in vitro data accumulated from a range of transgenic mouse models and iPSC-derived cardiomyocyte studies have demonstrated that deletion or mutation of *MYBPC3* causes HCM by disrupting the regulation of actomyosin, calcium homeostasis, and energy metabolism. In particular, *MYBPC3* mutations result in disorganized myosin movement, reduced calcium sensitivity, and elevated energy utilization, all of which contribute to the development of HCM^46,103,104^. N-terminal cMyBP-C protein (C0-C1 domain) is also cleaved after acute cardiac injury, such as myocardial infarction. Furthermore, this fragment has been shown to exert cytotoxic effects on cardiomyocyte biology, and as such, it can be used as an early indicator of cardiac injury when detected in the circulation^105,106^.

In contrast to *MYBPC3*, the pathogenic involvement of skeletal MyBP-C mutations was only recently revealed when *MYBPC1* mutations were found in patients with the congenital muscle diseases DA and LCCS. In 2010, Gurnet et al. pioneered the identification of two missense mutations of *MYBPC1* in two family members causing the W236R and Y856H amino acid substitutions^24^. The affected individuals suffer from distal arthrogryposis type 1 (DA1) featuring bilateral clubfoot and camptodactyly with ulnar deviation of the fingers. Since then, more than 10 unique *MYBPC1* mutations have been reported to be associated with the development of DA1, DA2 and LCCS^25^. In a recent publication, Geist et al.^107^ also showed that the knock-in E248K mutation of *MYBPC1* causes severe myopathy characterized by whole-body tremor, kyphosis, functional impairment and muscle atrophy. Similar to cMyBP-C, sMyBP-C also has multiple phosphorylation sites in the N-terminal region targeted by PKA and PKC^26^. The levels of sMyBP-C phosphorylation are increased after repeated muscle contractions but are decreased in aged and diseased muscles^108,109^. Moreover, according to a recent report, even transfecting the CRISPR‒Cas9 plasmid with electroporation to knock down 70% of sMyBP-C in adult skeletal muscle causes a significant reduction in peak force generation and the speed of contraction and relaxation. A disoriented sarcomere structure, a stretched Z-disk and a reduced sarcomere length were also observed in this skeletal muscle knockdown study^28^.

Moreover, two heterozygous compound *MYBPC2* mutations (T236I and S255T) were found in Turkish-origin family members with unclassified DA^110^. Few additional pathogenic *MYBPC2* mutations (V307A, F510fs, and A1065V) have been reported, but these remain to be completely characterized (Table 1). Unlike sMyBP-C, no study has reported a phosphorylation site on fMyBP-C. Our recent study, which used the first global *Mybpc2*-KO mouse model, has demonstrated that fMyBP-C is specifically expressed in fast twitch fibers, type2b and some type2x and is required for maximum force generation in the extensor digitorum longus. Elevated muscle damage and reduced calcium sensitivity are observed in KO muscle concurrent with a disrupted sarcomere microstructure.^27^

## Future directions

The general view holds that cMyBP-C predominates in the heart. However, sMyBP-C is expressed in both slow and fast muscle, and fMyBP-C is enriched in fast skeletal muscle, but low levels also present in slow skeletal muscle and heart muscle. An increasing body of the literature provides a more nuanced picture since the expression profile of MyBP-C paralogs becomes more complex when viewed through the prism of development, adulthood, and development of HF. Sarcomeric proteins play critical, but variable, roles based on their distinct functional properties. cMyBP-C clearly acts as a viscous load by tethering myosin to regulate the rate of cross-bridges and speed of contraction^86,111–113^. Moreover, a significant gap in knowledge accompanies the additional role of skeletal paralogs in slow or fast subtypes of skeletal muscle development and growth as well as their regulatory role(s) in cardiac muscle. We now have a number of novel *Mybpc1* and *Mybpc2* mouse models, including global and conditional KO mouse lines for sMyBP-C and fMyBP-C, as well as exogenous methods to overexpress these proteins in both skeletal muscle and heart. In addition, we have the ability to carefully interrogate the specific domain regions of sMyBP-C and fMyBP-C by utilizing various in vitro systems to define actomyosin interactions based on comparisons with those of cMyBP-C. Thus, a significant goal of future studies focuses on defining the regulatory roles of sMyBP-C and fMyBP-C in contractility.

## Concluding remarks

**Fig. 4:**
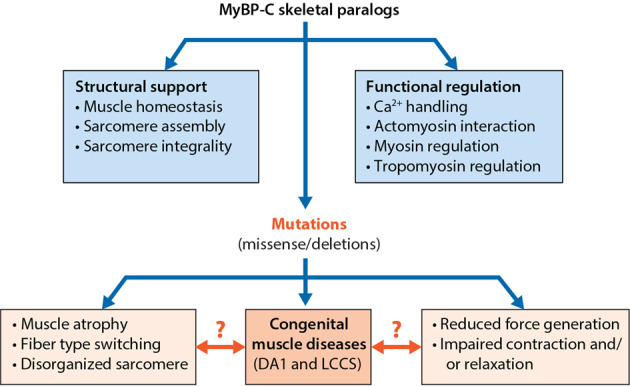
Schematic diagram of two major functions of skeletal MyBP-C protein and pathogenic consequences by their deletion and mutations.

In summary, the MyBP-C family consists of sarcomeric thick filament proteins essential for normal sarcomere structure and function in cardiac and skeletal muscle. All three MyBP-C paralogs, slow (sMyBP-C), fast (fMyBP-C) and cardiac (cMyBP-C), are encoded by separate genes. Recent studies have established that the cardiomyocyte-specific cMyBP-C is a critical trans-filament protein that acts as a bridge between thick and thin filament proteins via its N’-region. Importantly, mutations in cMyBP-C paralogs are the most common cause of cardiomyopathies. Conversely, sMyBP-C and fMyBP-C are predominantly found in skeletal muscles, and mutations in these two isoforms are known to be associated with skeletal muscle diseases (Fig. 4). To date, a systematic study to determine the specific role of each paralog in skeletal myocytes and cardiomyocytes has yet to be conducted. Several outstanding questions challenge the current dogma regarding the tissue-specific expression and functional relevance of these isoforms; hence, further essential investigations are being conducted to elucidate the potential of modulating these proteins by performing gene therapy as a therapeutic approach.
